# *cis*-3-(*tert*-Butoxy­carbonyl­amino)cyclo­hexa­necarboxylic acid

**DOI:** 10.1107/S1600536808027098

**Published:** 2008-08-30

**Authors:** Yu Hu, XiaoXia Sun, Ying Guo, Hua Yao

**Affiliations:** a Experimental Chemistry Center, Nanchang University, Nanchang 330031, People’s Republic of China; b Jiangxi Key Laboratory of Organic Chemistry, Jiangxi Science and Technology Normal University, Nanchang 330013, People’s Republic of China

## Abstract

The title compound, C_12_H_21_NO_4_, a γ-aminobutyric acid derivative, crystallizes with two mol­ecules in the asymmetric unit. The crystal structure is stabilized by inter­molecular N—H⋯O and O—H⋯O hydrogen bonds, forming a strand. An intramolecular N—H⋯O hydrogen bond is also observed.

## Related literature

For related literature, see: Allan *et al.* (1981 ▶); Amorin *et al.* (2003 ▶); Hu *et al.* (2006 ▶); Roberts *et al.* (1976 ▶); Schousboe (2000 ▶).
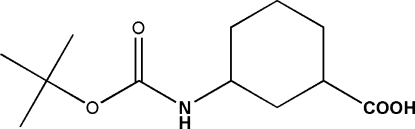


## Experimental

### 

#### Crystal data

C_12_H_21_NO_4_
*M* *_r_* = 243.30
Triclinic, 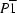

*a* = 5.854 (1) Å
*b* = 10.000 (2) Å
*c* = 23.014 (5) Å
α = 85.64 (2)°
β = 88.68 (2)°
γ = 88.51 (2)°
*V* = 1342.6 (4) Å^3^
*Z* = 4
Mo *K*α radiation
μ = 0.09 mm^−1^
*T* = 296 (2) K
0.56 × 0.46 × 0.20 mm

#### Data collection

Bruker SMART 1K area-detector diffractometer
Absorption correction: multi-scan (*SADABS*; Sheldrick, 1996 ▶) *T*_min_ = 0.767, *T*_max_ = 0.921 (expected range = 0.818–0.982)
5514 measured reflections
4822 independent reflections
2483 reflections with *I* > 2σ(*I*)
*R*_int_ = 0.017

#### Refinement

*R*[*F* ^2^ > 2σ(*F* ^2^)] = 0.044
*wR*(*F* ^2^) = 0.109
*S* = 0.81
4822 reflections
324 parameters
2 restraints
H atoms treated by a mixture of independent and constrained refinement
Δρ_max_ = 0.22 e Å^−3^
Δρ_min_ = −0.17 e Å^−3^

### 

Data collection: *SMART* (Bruker, 1999 ▶); cell refinement: *SMART*; data reduction: *SAINT-Plus* (Bruker, 1999 ▶); program(s) used to solve structure: *SHELXS97* (Sheldrick, 2008 ▶); program(s) used to refine structure: *SHELXL97* (Sheldrick, 2008 ▶); molecular graphics: *SHELXL97*; software used to prepare material for publication: *SHELXTL* (Sheldrick, 2008 ▶).

## Supplementary Material

Crystal structure: contains datablocks I, global. DOI: 10.1107/S1600536808027098/bt2775sup1.cif

Structure factors: contains datablocks I. DOI: 10.1107/S1600536808027098/bt2775Isup2.hkl

Additional supplementary materials:  crystallographic information; 3D view; checkCIF report

## Figures and Tables

**Table 1:** Hydrogen-bond geometry (Å, °)

*D*—H⋯*A*	*D*—H	H⋯*A*	*D*⋯*A*	*D*—H⋯*A*
N1—H1*A*⋯O6	0.856 (9)	2.163 (10)	3.009 (2)	169 (2)
N2—H2*A*⋯O2^i^	0.854 (9)	2.180 (11)	3.013 (2)	164.9 (19)
O3—H3⋯O4^ii^	0.82	1.86	2.672 (2)	172
O7—H7⋯O8^iii^	0.82	1.84	2.656 (2)	171

Symmetry codes: (i) 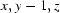; (ii) 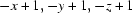; (iii) 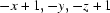.

**Fig. 1.**
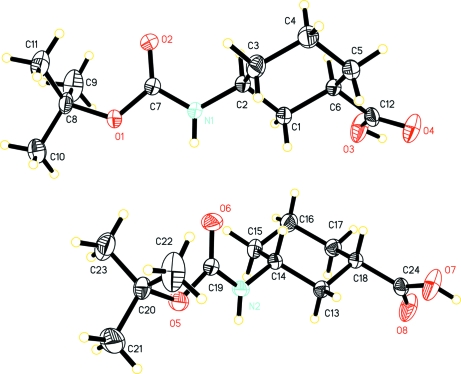
View of the two molecules in the asymmetric unit of the title compound, with anisotropic displacement parameters drawn at the 50% probability level.

**Fig. 2.**
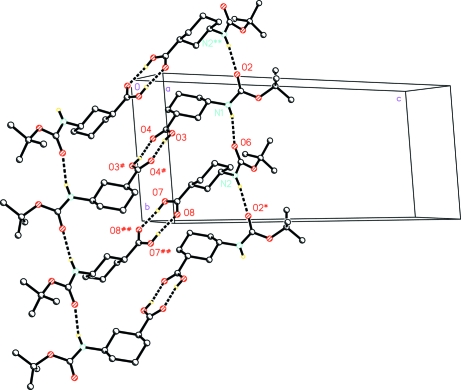
A view of the hydrogen-bonded strands (Dashed lines). The strands are aligned parallel to the crystallographic b axis. H atoms not involved in H-bonding have been omitted for clarity. Symmetry codes: (*) x,y+1,z; (**) x,y-1,z; (#) 1 - x,1 - y,-z; (##) 1 - x,2 - y,-z.

## supplementary crystallographic information

### Comment

γ aminobutyric acid (GABA) and its derivatives have an extensive appliction in medicine. For instance, GABA is an important inhibitory neurotransmitter in certain neurological and psychiatric disorders (Schousboe, 2000; Roberts *et al.*, 1976). As the analogue of GABA, *cis*-3-aminocyclohexanecarboxylic acid is an inhibitor of GABA uptake (Allan *et al.*, 1981). *cis*-3-aminocyclohexanecarboxylic acid is incorporated in cyclic peptides and organic nanotubes (Amorin *et al.*, 2003). The title compound  is a key intermediate   for the synthesis of *cis*-3- aminocyclohexanecarboxylic acid (Hu *et al.*, 2006). The synthesis and crystal structure of the title compound are described in this paper.

Interolecular O-H···O hydrogen bonds  result  in eight-membered rings that can be  described in terms of graph-set notation as *R_2_^2^(8)* (Figure 2).

One-dimensional  strands are formed along the crystallographic *b* axis by N-H···O hydrogen bonds (Figure 2 and Table 2).

### Experimental

*cis*-3-*tert*-butoxycarbonylamino-cyclohexanecarboxylic acid was synthesized from 3-aminobenzoic acid (Amorin *et al.*, 2003). The compound identity was conformed by the NMR spectra and IR. Crystal   were obtained from ethyl acetate by solvent evaporation. ^1^H NMR in CDCl_3_ (300 MHz): 9.20–10.4 (br, 1H,), 5.72 (s, 1H), 4.82 (s, H), 3.67–3.74 (m, 1H), 1.43 (s, 9H), 1.05–2.40 (m, 8H).

### Refinement

H atoms bonded to C and O were geometrically positioned and   treated as riding on their parent C atoms, with C—H distances in the range of 0.82–0.98 Å, with *U*_iso_ (H) = 1.2–1.5 times *U*_eq_ of the parent atom. H atoms attached to N1 and N2 were located in difference Fourier maps and refined initially with the N-H distance restrained to 0.86 Å.

### Crystal data

**Table:**

C_12_H_21_NO_4_	*Z* = 4
*M**_r_* = 243.30	*F*_000_ = 528
Triclinic, *P*1	*D*_x_ = 1.204 Mg m^−^^3^
Hall symbol: -P 1	Melting point: 409 K
*a* = 5.854 (1) Å	Mo *K*α radiation λ = 0.71073 Å
*b* = 10.000 (2) Å	Cell parameters from 29 reflections
*c* = 23.014 (5) Å	θ = 4.1–13.9º
α = 85.64 (2)º	µ = 0.09 mm^−^^1^
β = 88.68 (2)º	*T* = 296 (2) K
γ = 88.51 (2)º	Block, colorless
*V* = 1342.6 (4) Å^3^	0.56 × 0.46 × 0.20 mm

### Data collection

**Table:**

Bruker SMART 1K area-detector diffractometer	4822 independent reflections
Radiation source: fine-focus sealed tube	2483 reflections with *I* > 2σ(*I*)
Monochromator: graphite	*R*_int_ = 0.017
*T* = 293(2) K	θ_max_ = 25.3º
phi and ω scans	θ_min_ = 1.8º
Absorption correction: multi-scan(SADABS; Sheldrick, 1996)	*h* = 0→7
*T*_min_ = 0.767, *T*_max_ = 0.921	*k* = −11→11
5514 measured reflections	*l* = −27→27

### Refinement

**Table:**

Refinement on *F*^2^	Secondary atom site location: difference Fourier map
Least-squares matrix: full	Hydrogen site location: inferred from neighbouring sites
*R*[*F*^2^ > 2σ(*F*^2^)] = 0.044	H atoms treated by a mixture of independent and constrained refinement
*wR*(*F*^2^) = 0.109	*w* = 1/[σ^2^(*F*_o_^2^) + (0.0564*P*)^2^] where *P* = (*F*_o_^2^ + 2*F*_c_^2^)/3
*S* = 0.81	(Δ/σ)_max_ < 0.001
4822 reflections	Δρ_max_ = 0.22 e Å^−^^3^
324 parameters	Δρ_min_ = −0.17 e Å^−^^3^
2 restraints	Extinction correction: SHELXL, Fc^*^=kFc[1+0.001xFc^2^λ^3^/sin(2θ)]^-1/4^
Primary atom site location: structure-invariant direct methods	Extinction coefficient: 0.0098 (13)

### Special details

**Table:**

Geometry. All e.s.d.'s (except the e.s.d. in the dihedral angle between two l.s. planes) are estimated using the full covariance matrix. The cell e.s.d.'s are taken into account individually in the estimation of e.s.d.'s in distances, angles and torsion angles; correlations between e.s.d.'s in cell parameters are only used when they are defined by crystal symmetry. An approximate (isotropic) treatment of cell e.s.d.'s is used for estimating e.s.d.'s involving l.s. planes.
Refinement. Refinement of *F*^2^ against ALL reflections. The weighted *R*-factor *wR* and goodness of fit *S* are based on *F*^2^, conventional *R*-factors *R* are based on *F*, with *F* set to zero for negative *F*^2^. The threshold expression of *F*^2^ > σ(*F*^2^) is used only for calculating *R*-factors(gt) *etc*. and is not relevant to the choice of reflections for refinement. *R*-factors based on *F*^2^ are statistically about twice as large as those based on *F*, and *R*- factors based on ALL data will be even larger.

### Fractional atomic coordinates and isotropic or equivalent isotropic displacement parameters (Å^2^)

**Table:**

	*x*	*y*	*z*	*U*_iso_*/*U*_eq_	
O1	0.6276 (3)	0.87356 (14)	0.15126 (6)	0.0548 (5)	
O2	0.5946 (3)	1.03347 (15)	0.21599 (7)	0.0620 (5)	
O3	0.6134 (3)	0.64184 (19)	0.45997 (9)	0.0823 (6)	
H3	0.6355	0.5778	0.4838	0.099*	
O4	0.2737 (3)	0.55582 (18)	0.46040 (8)	0.0797 (6)	
N1	0.4694 (4)	0.82327 (18)	0.23756 (7)	0.0481 (5)	
C1	0.4318 (4)	0.7291 (2)	0.33857 (8)	0.0408 (5)	
H1C	0.5960	0.7136	0.3392	0.049*	
H1B	0.3623	0.6490	0.3265	0.049*	
C2	0.3427 (4)	0.7572 (2)	0.39994 (8)	0.0384 (5)	
H2	0.4183	0.8372	0.4113	0.046*	
C3	0.0867 (4)	0.7873 (2)	0.39991 (9)	0.0467 (6)	
H3A	0.0069	0.7080	0.3906	0.056*	
H3B	0.0358	0.8099	0.4384	0.056*	
C4	0.0288 (4)	0.9027 (2)	0.35566 (10)	0.0525 (6)	
H4A	0.0938	0.9843	0.3677	0.063*	
H4B	−0.1358	0.9160	0.3546	0.063*	
C5	0.1199 (4)	0.8768 (2)	0.29489 (9)	0.0536 (6)	
H5A	0.0424	0.8011	0.2810	0.064*	
H5B	0.0880	0.9547	0.2684	0.064*	
C6	0.3752 (4)	0.8474 (2)	0.29527 (8)	0.0403 (5)	
H6	0.4493	0.9263	0.3083	0.048*	
C7	0.5654 (4)	0.9200 (2)	0.20301 (9)	0.0437 (6)	
C8	0.7620 (4)	0.9548 (2)	0.10769 (9)	0.0483 (6)	
C9	0.6330 (5)	1.0795 (3)	0.08659 (11)	0.0732 (8)	
H9A	0.4824	1.0567	0.0756	0.088*	
H9B	0.7120	1.1221	0.0535	0.088*	
H9C	0.6216	1.1397	0.1171	0.088*	
C10	0.7906 (6)	0.8626 (3)	0.05895 (11)	0.0877 (10)	
H10A	0.8705	0.7819	0.0729	0.105*	
H10B	0.8766	0.9066	0.0273	0.105*	
H10C	0.6430	0.8408	0.0456	0.105*	
C11	0.9874 (5)	0.9845 (3)	0.13189 (13)	0.0946 (11)	
H11A	0.9663	1.0492	0.1604	0.114*	
H11B	1.0866	1.0200	0.1010	0.114*	
H11C	1.0546	0.9036	0.1499	0.114*	
C12	0.4076 (4)	0.6421 (2)	0.44293 (9)	0.0414 (5)	
O5	0.3565 (3)	0.37430 (14)	0.15151 (6)	0.0550 (5)	
O6	0.3842 (3)	0.53682 (15)	0.21453 (6)	0.0597 (5)	
O7	0.3647 (3)	0.1116 (2)	0.44631 (8)	0.0765 (6)	
H7	0.3445	0.0571	0.4743	0.092*	
O8	0.7245 (3)	0.08103 (17)	0.47100 (7)	0.0692 (5)	
N2	0.5105 (3)	0.32454 (17)	0.23698 (7)	0.0458 (5)	
C13	0.5512 (4)	0.2287 (2)	0.33700 (8)	0.0401 (5)	
H13A	0.6327	0.1504	0.3240	0.048*	
H13B	0.3892	0.2102	0.3385	0.048*	
C14	0.6306 (4)	0.2564 (2)	0.39808 (8)	0.0382 (5)	
H14	0.5424	0.3347	0.4100	0.046*	
C15	0.8821 (4)	0.2927 (2)	0.39721 (9)	0.0477 (6)	
H15A	0.9757	0.2159	0.3873	0.057*	
H15B	0.9243	0.3159	0.4356	0.057*	
C16	0.9262 (4)	0.4108 (2)	0.35278 (10)	0.0543 (6)	
H16A	0.8474	0.4902	0.3656	0.065*	
H16B	1.0886	0.4280	0.3507	0.065*	
C17	0.8453 (4)	0.3842 (2)	0.29241 (9)	0.0536 (6)	
H17A	0.8678	0.4634	0.2660	0.064*	
H17B	0.9358	0.3110	0.2777	0.064*	
C18	0.5954 (4)	0.3489 (2)	0.29415 (8)	0.0395 (5)	
H18	0.5081	0.4256	0.3080	0.047*	
C19	0.4147 (4)	0.4221 (2)	0.20222 (9)	0.0426 (6)	
C20	0.2250 (4)	0.4572 (2)	0.10807 (9)	0.0482 (6)	
C21	0.3591 (5)	0.5763 (3)	0.08456 (11)	0.0705 (8)	
H21A	0.3771	0.6359	0.1148	0.085*	
H21B	0.2791	0.6225	0.0528	0.085*	
H21C	0.5069	0.5465	0.0710	0.085*	
C22	0.1969 (6)	0.3620 (3)	0.06073 (11)	0.0893 (10)	
H22A	0.3447	0.3347	0.0463	0.107*	
H22B	0.1117	0.4065	0.0294	0.107*	
H22C	0.1164	0.2845	0.0764	0.107*	
C23	−0.0011 (5)	0.4961 (3)	0.13346 (12)	0.0863 (10)	
H23A	−0.0716	0.4182	0.1522	0.104*	
H23B	−0.0972	0.5346	0.1030	0.104*	
H23C	0.0198	0.5607	0.1616	0.104*	
C24	0.5776 (4)	0.1408 (2)	0.44197 (9)	0.0426 (6)	
H1A	0.452 (4)	0.7445 (12)	0.2264 (9)	0.048 (7)*	
H2A	0.522 (3)	0.2460 (12)	0.2249 (8)	0.039 (6)*	

### Atomic displacement parameters (Å^2^)

**Table:**

	*U*^11^	*U*^22^	*U*^33^	*U*^12^	*U*^13^	*U*^23^
O1	0.0864 (13)	0.0413 (9)	0.0361 (9)	−0.0087 (8)	0.0145 (8)	−0.0006 (7)
O2	0.1046 (15)	0.0365 (9)	0.0447 (10)	−0.0099 (9)	0.0104 (9)	−0.0030 (8)
O3	0.0604 (13)	0.0895 (15)	0.0894 (15)	−0.0081 (11)	−0.0195 (11)	0.0498 (11)
O4	0.0745 (14)	0.0736 (13)	0.0854 (14)	−0.0219 (11)	−0.0229 (10)	0.0437 (11)
N1	0.0844 (16)	0.0307 (11)	0.0288 (10)	−0.0057 (10)	0.0068 (9)	−0.0015 (9)
C1	0.0493 (14)	0.0388 (12)	0.0334 (12)	0.0036 (10)	0.0013 (10)	0.0006 (9)
C2	0.0499 (14)	0.0344 (11)	0.0305 (11)	−0.0003 (10)	−0.0030 (10)	0.0003 (9)
C3	0.0507 (15)	0.0479 (14)	0.0408 (13)	0.0026 (11)	0.0020 (11)	−0.0007 (11)
C4	0.0536 (16)	0.0525 (14)	0.0504 (14)	0.0098 (12)	−0.0034 (12)	0.0003 (12)
C5	0.0667 (18)	0.0530 (15)	0.0399 (14)	0.0098 (13)	−0.0113 (12)	0.0041 (11)
C6	0.0584 (16)	0.0356 (12)	0.0266 (11)	0.0001 (11)	−0.0007 (10)	−0.0006 (9)
C7	0.0648 (17)	0.0345 (13)	0.0313 (12)	0.0029 (11)	−0.0034 (11)	0.0006 (10)
C8	0.0589 (16)	0.0491 (14)	0.0350 (12)	−0.0040 (12)	0.0051 (11)	0.0082 (11)
C9	0.087 (2)	0.0726 (18)	0.0554 (16)	0.0072 (16)	0.0048 (15)	0.0203 (14)
C10	0.139 (3)	0.073 (2)	0.0496 (17)	−0.0045 (19)	0.0331 (17)	−0.0041 (15)
C11	0.066 (2)	0.142 (3)	0.071 (2)	−0.012 (2)	−0.0066 (16)	0.028 (2)
C12	0.0492 (16)	0.0454 (14)	0.0294 (12)	−0.0024 (12)	−0.0003 (11)	−0.0003 (10)
O5	0.0900 (13)	0.0407 (9)	0.0344 (9)	0.0082 (8)	−0.0216 (8)	−0.0015 (7)
O6	0.1010 (14)	0.0325 (9)	0.0460 (10)	0.0044 (9)	−0.0174 (9)	−0.0025 (7)
O7	0.0646 (13)	0.0912 (15)	0.0673 (13)	−0.0167 (11)	−0.0103 (10)	0.0429 (10)
O8	0.0616 (12)	0.0802 (12)	0.0598 (11)	0.0021 (10)	−0.0067 (9)	0.0342 (10)
N2	0.0777 (15)	0.0305 (11)	0.0291 (10)	0.0044 (10)	−0.0097 (9)	−0.0011 (9)
C13	0.0491 (14)	0.0377 (12)	0.0334 (12)	−0.0032 (10)	−0.0063 (10)	0.0011 (9)
C14	0.0526 (15)	0.0335 (11)	0.0281 (11)	−0.0002 (10)	−0.0027 (10)	0.0006 (9)
C15	0.0576 (16)	0.0470 (14)	0.0385 (13)	−0.0052 (11)	−0.0095 (11)	0.0003 (10)
C16	0.0569 (16)	0.0544 (15)	0.0516 (15)	−0.0155 (12)	−0.0063 (12)	0.0035 (12)
C17	0.0651 (18)	0.0549 (15)	0.0389 (13)	−0.0094 (13)	0.0045 (12)	0.0093 (11)
C18	0.0570 (15)	0.0332 (11)	0.0281 (11)	0.0003 (10)	−0.0029 (10)	−0.0002 (9)
C19	0.0587 (16)	0.0353 (13)	0.0333 (12)	−0.0016 (11)	−0.0043 (11)	0.0008 (10)
C20	0.0588 (16)	0.0518 (14)	0.0322 (12)	0.0036 (12)	−0.0077 (11)	0.0098 (11)
C21	0.078 (2)	0.0773 (19)	0.0532 (16)	−0.0065 (16)	−0.0084 (14)	0.0206 (14)
C22	0.138 (3)	0.076 (2)	0.0557 (18)	0.0055 (19)	−0.0464 (18)	−0.0007 (15)
C23	0.063 (2)	0.125 (3)	0.0653 (19)	0.0061 (18)	−0.0004 (15)	0.0247 (18)
C24	0.0528 (16)	0.0483 (14)	0.0265 (11)	−0.0041 (12)	−0.0032 (11)	−0.0007 (10)

### Geometric parameters (Å, °)

**Table:**

O1—C7	1.350 (2)	O5—C19	1.349 (2)
O1—C8	1.468 (2)	O5—C20	1.468 (2)
O2—C7	1.212 (2)	O6—C19	1.210 (2)
O3—C12	1.276 (3)	O7—C24	1.288 (3)
O3—H3	0.8200	O7—H7	0.8200
O4—C12	1.220 (2)	O8—C24	1.217 (2)
N1—C7	1.332 (3)	N2—C19	1.334 (3)
N1—C6	1.461 (3)	N2—C18	1.457 (3)
N1—H1A	0.856 (9)	N2—H2A	0.854 (9)
C1—C6	1.522 (3)	C13—C18	1.519 (3)
C1—C2	1.538 (3)	C13—C14	1.538 (3)
C1—H1C	0.9700	C13—H13A	0.9700
C1—H1B	0.9700	C13—H13B	0.9700
C2—C12	1.507 (3)	C14—C24	1.510 (3)
C2—C3	1.521 (3)	C14—C15	1.525 (3)
C2—H2	0.9800	C14—H14	0.9800
C3—C4	1.517 (3)	C15—C16	1.526 (3)
C3—H3A	0.9700	C15—H15A	0.9700
C3—H3B	0.9700	C15—H15B	0.9700
C4—C5	1.524 (3)	C16—C17	1.522 (3)
C4—H4A	0.9700	C16—H16A	0.9700
C4—H4B	0.9700	C16—H16B	0.9700
C5—C6	1.515 (3)	C17—C18	1.513 (3)
C5—H5A	0.9700	C17—H17A	0.9700
C5—H5B	0.9700	C17—H17B	0.9700
C6—H6	0.9800	C18—H18	0.9800
C8—C11	1.490 (3)	C20—C23	1.489 (3)
C8—C9	1.496 (3)	C20—C21	1.503 (3)
C8—C10	1.509 (3)	C20—C22	1.515 (3)
C9—H9A	0.9600	C21—H21A	0.9600
C9—H9B	0.9600	C21—H21B	0.9600
C9—H9C	0.9600	C21—H21C	0.9600
C10—H10A	0.9600	C22—H22A	0.9600
C10—H10B	0.9600	C22—H22B	0.9600
C10—H10C	0.9600	C22—H22C	0.9600
C11—H11A	0.9600	C23—H23A	0.9600
C11—H11B	0.9600	C23—H23B	0.9600
C11—H11C	0.9600	C23—H23C	0.9600
			
C7—O1—C8	121.42 (16)	C19—O5—C20	121.23 (16)
C12—O3—H3	109.5	C24—O7—H7	109.5
C7—N1—C6	122.03 (18)	C19—N2—C18	121.92 (17)
C7—N1—H1A	122.0 (15)	C19—N2—H2A	118.2 (14)
C6—N1—H1A	115.9 (15)	C18—N2—H2A	119.8 (14)
C6—C1—C2	110.34 (16)	C18—C13—C14	110.31 (16)
C6—C1—H1C	109.6	C18—C13—H13A	109.6
C2—C1—H1C	109.6	C14—C13—H13A	109.6
C6—C1—H1B	109.6	C18—C13—H13B	109.6
C2—C1—H1B	109.6	C14—C13—H13B	109.6
H1C—C1—H1B	108.1	H13A—C13—H13B	108.1
C12—C2—C3	112.57 (18)	C24—C14—C15	112.45 (18)
C12—C2—C1	109.99 (16)	C24—C14—C13	111.16 (16)
C3—C2—C1	110.88 (17)	C15—C14—C13	111.45 (17)
C12—C2—H2	107.7	C24—C14—H14	107.2
C3—C2—H2	107.7	C15—C14—H14	107.2
C1—C2—H2	107.7	C13—C14—H14	107.2
C4—C3—C2	110.80 (18)	C14—C15—C16	110.42 (18)
C4—C3—H3A	109.5	C14—C15—H15A	109.6
C2—C3—H3A	109.5	C16—C15—H15A	109.6
C4—C3—H3B	109.5	C14—C15—H15B	109.6
C2—C3—H3B	109.5	C16—C15—H15B	109.6
H3A—C3—H3B	108.1	H15A—C15—H15B	108.1
C3—C4—C5	111.78 (18)	C17—C16—C15	111.92 (18)
C3—C4—H4A	109.3	C17—C16—H16A	109.2
C5—C4—H4A	109.3	C15—C16—H16A	109.2
C3—C4—H4B	109.3	C17—C16—H16B	109.2
C5—C4—H4B	109.3	C15—C16—H16B	109.2
H4A—C4—H4B	107.9	H16A—C16—H16B	107.9
C6—C5—C4	111.06 (18)	C18—C17—C16	111.07 (18)
C6—C5—H5A	109.4	C18—C17—H17A	109.4
C4—C5—H5A	109.4	C16—C17—H17A	109.4
C6—C5—H5B	109.4	C18—C17—H17B	109.4
C4—C5—H5B	109.4	C16—C17—H17B	109.4
H5A—C5—H5B	108.0	H17A—C17—H17B	108.0
N1—C6—C5	112.69 (18)	N2—C18—C17	112.74 (18)
N1—C6—C1	110.26 (16)	N2—C18—C13	110.10 (16)
C5—C6—C1	110.66 (18)	C17—C18—C13	111.08 (17)
N1—C6—H6	107.7	N2—C18—H18	107.6
C5—C6—H6	107.7	C17—C18—H18	107.6
C1—C6—H6	107.7	C13—C18—H18	107.6
O2—C7—N1	125.2 (2)	O6—C19—N2	124.8 (2)
O2—C7—O1	124.5 (2)	O6—C19—O5	125.00 (19)
N1—C7—O1	110.26 (18)	N2—C19—O5	110.16 (18)
O1—C8—C11	109.95 (19)	O5—C20—C23	110.03 (18)
O1—C8—C9	111.3 (2)	O5—C20—C21	110.79 (19)
C11—C8—C9	112.2 (2)	C23—C20—C21	112.6 (2)
O1—C8—C10	102.23 (18)	O5—C20—C22	102.20 (18)
C11—C8—C10	111.0 (2)	C23—C20—C22	110.8 (2)
C9—C8—C10	109.7 (2)	C21—C20—C22	109.9 (2)
C8—C9—H9A	109.5	C20—C21—H21A	109.5
C8—C9—H9B	109.5	C20—C21—H21B	109.5
H9A—C9—H9B	109.5	H21A—C21—H21B	109.5
C8—C9—H9C	109.5	C20—C21—H21C	109.5
H9A—C9—H9C	109.5	H21A—C21—H21C	109.5
H9B—C9—H9C	109.5	H21B—C21—H21C	109.5
C8—C10—H10A	109.5	C20—C22—H22A	109.5
C8—C10—H10B	109.5	C20—C22—H22B	109.5
H10A—C10—H10B	109.5	H22A—C22—H22B	109.5
C8—C10—H10C	109.5	C20—C22—H22C	109.5
H10A—C10—H10C	109.5	H22A—C22—H22C	109.5
H10B—C10—H10C	109.5	H22B—C22—H22C	109.5
C8—C11—H11A	109.5	C20—C23—H23A	109.5
C8—C11—H11B	109.5	C20—C23—H23B	109.5
H11A—C11—H11B	109.5	H23A—C23—H23B	109.5
C8—C11—H11C	109.5	C20—C23—H23C	109.5
H11A—C11—H11C	109.5	H23A—C23—H23C	109.5
H11B—C11—H11C	109.5	H23B—C23—H23C	109.5
O4—C12—O3	122.4 (2)	O8—C24—O7	123.0 (2)
O4—C12—C2	122.7 (2)	O8—C24—C14	122.6 (2)
O3—C12—C2	114.9 (2)	O7—C24—C14	114.4 (2)
			
C6—C1—C2—C12	−177.75 (18)	C18—C13—C14—C24	177.21 (18)
C6—C1—C2—C3	57.1 (2)	C18—C13—C14—C15	−56.5 (2)
C12—C2—C3—C4	−179.43 (18)	C24—C14—C15—C16	−179.33 (18)
C1—C2—C3—C4	−55.7 (2)	C13—C14—C15—C16	55.1 (2)
C2—C3—C4—C5	55.1 (3)	C14—C15—C16—C17	−54.7 (3)
C3—C4—C5—C6	−55.6 (3)	C15—C16—C17—C18	55.7 (3)
C7—N1—C6—C5	95.8 (3)	C19—N2—C18—C17	−91.8 (3)
C7—N1—C6—C1	−140.0 (2)	C19—N2—C18—C13	143.5 (2)
C4—C5—C6—N1	−179.42 (18)	C16—C17—C18—N2	179.19 (17)
C4—C5—C6—C1	56.6 (2)	C16—C17—C18—C13	−56.7 (2)
C2—C1—C6—N1	177.30 (18)	C14—C13—C18—N2	−177.45 (17)
C2—C1—C6—C5	−57.4 (2)	C14—C13—C18—C17	56.9 (2)
C6—N1—C7—O2	3.7 (4)	C18—N2—C19—O6	−1.7 (4)
C6—N1—C7—O1	−176.82 (19)	C18—N2—C19—O5	178.42 (19)
C8—O1—C7—O2	6.9 (3)	C20—O5—C19—O6	−6.7 (3)
C8—O1—C7—N1	−172.59 (19)	C20—O5—C19—N2	173.15 (19)
C7—O1—C8—C11	61.5 (3)	C19—O5—C20—C23	−60.9 (3)
C7—O1—C8—C9	−63.5 (3)	C19—O5—C20—C21	64.3 (3)
C7—O1—C8—C10	179.4 (2)	C19—O5—C20—C22	−178.6 (2)
C3—C2—C12—O4	23.3 (3)	C15—C14—C24—O8	−3.1 (3)
C1—C2—C12—O4	−100.9 (3)	C13—C14—C24—O8	122.6 (2)
C3—C2—C12—O3	−156.4 (2)	C15—C14—C24—O7	176.5 (2)
C1—C2—C12—O3	79.5 (3)	C13—C14—C24—O7	−57.8 (3)

### Hydrogen-bond geometry (Å, °)

**Table:**

*D*—H···*A*	*D*—H	H···*A*	*D*···*A*	*D*—H···*A*
N1—H1A···O6	0.856 (9)	2.163 (10)	3.009 (2)	169 (2)
N2—H2A···O2^i^	0.854 (9)	2.180 (11)	3.013 (2)	164.9 (19)
O3—H3···O4^ii^	0.82	1.86	2.672 (2)	172
O7—H7···O8^iii^	0.82	1.84	2.656 (2)	171

Symmetry codes: (i) *x*, *y*−1, *z*; (ii) −*x*+1, −*y*+1, −*z*+1; (iii) −*x*+1, −*y*, −*z*+1.
